# Thermoresponsive Dual-Structured Gel Emulsions Stabilized by Glycyrrhizic Acid Nanofibrils in Combination with Monoglyceride Crystals

**DOI:** 10.3390/molecules27196542

**Published:** 2022-10-03

**Authors:** Jialing Chen, Qing Li, Ruijie Du, Xinke Yu, Zhili Wan, Xiaoquan Yang

**Affiliations:** 1 Laboratory of Food Proteins and Colloids, School of Food Science and Engineering, Guangdong Province Key Laboratory for Green Processing of Natural Products and Product Safety, South China University of Technology, Guangzhou 510640, China; 2 Overseas Expertise Introduction Center for Discipline Innovation of Food Nutrition and Human Health (111 Center), Guangzhou 510640, China

**Keywords:** glycyrrhizic acid, monoglyceride, dual-structured gel emulsions, thermoresponsive, controlled release

## Abstract

Responsive dual-structured emulsions and gel emulsions have attracted more and more attention due to their complex microstructures, on-demand responsive properties, and controlled release of active cargoes. In this work, the effect of monoglyceride (MG)-based oil phase structuring on the formation and stability, structural properties, and thermoresponsive and cargo release behavior of gel emulsions stabilized by glycyrrhizic acid (GA) nanofibrils were investigated. Owing to the formation of GA fibrillar networks in the aqueous phase and MG crystalline networks in the oil phase, a stable dual-structured gel emulsion can be successfully developed. The microstructure of the dual-structured gel emulsions largely depended on the concentration of MG in the oil phase. At low MG concentrations (1–2 wt%), the larger formed and lamellar MG crystals may pierce the interfacial fibrillar film, inducing the formation of partially coalesced droplets. In contrast, at high MG concentrations (4 wt% or above), the smaller MG crystals with enhanced interfacial activity can lead to the formation of a bilayer shell of GA nanofibrils and MG crystals, thus efficiently inhibiting the interfacial film damage and forming a jamming structure with homogeneously distributed small droplets. Compared to pure GA nanofibril gel emulsions, the GA−MG dual-structured gel emulsions showed significantly improved mechanical performance as well as good thermoresponsive behavior. Moreover, these stable GA−MG gel emulsions can be used as food-grade delivery vehicles for encapsulating and protecting hydrophobic and hydrophilic bioactive cargoes. They also have great potential as novel and efficient aroma delivery systems showing highly controlled volatile release. The dual-structured emulsion strategy is expected to broaden the applications of natural saponin GA-based gel emulsions in the food, pharmaceutical, and personal care industries.

## 1. Introduction

Gel emulsions, also known as emulsion gels, are complex, structured, multiphase, soft materials with excellent physical stability and viscoelastic mechanical properties. In gel emulsions, the emulsified droplets are entrapped within a gel matrix, thus preventing droplet flocculation and coalescence [1]. Recently, the development of novel gel emulsions has attracted more interest in the food industry to meet the increasing demand for saturated fat replacement and/or fat mimetics in meat alternatives based on health and nutritional considerations [2,3]. Depending on the gelation mechanism of gel emulsions, they can be mainly divided into four classes: water continuous emulsions with oil droplets trapped in gelled aqueous solution [4], oil continuous gel emulsions containing water droplets [5,6], emulsions with both gelled water and oil phases (i.e., dual-structured gel emulsions) [7,8], and highly concentrated emulsions in which a continuous network is formed by the tight stacking of the oil droplets [3,9]. Among these structured emulsions, the dual-structured gel emulsions possess excellent mechanical strength, better physical stability, more complex and unique microstructures including O/W, W/O, and bi-continuous droplet structures, and a greater capacity to encapsulate and release bioactive ingredients [8,10,11,12]. For example, Zheng et al. reported that food-grade dual-structured emulsions based on κ-carrageenan hydrogel and monoglyceride oleogels can enhance the light and thermal stability of *β*-carotene with increasing oleogel fraction [13]. Rehman et al. designed the carbopol and beeswax dual-structured emulsions for the delivery of imiquimod and found that they have a higher ability to control imiquimod-induced inflammatory side effects in the treatment of skin cancer [14]. All these features of dual-structured emulsions enable them to have the great potential to fabricate novel formulations and products with diverse applications in healthy food, cosmetics, and pharmaceutical fields [2,9].

We recently found that the naturally occurring triterpenoid saponin glycyrrhizic acid (GA) can be used as the sole stabilizer to make a new class of food-grade oil-in-water (O/W) gel emulsions [15,16,17]. GA, a main active ingredient of licorice root extract, possesses a wide range of biological effects, such as antitumor, antivirus, anti-inflammatory, and antifungal activities [18]. In food processing, GA has been widely applied as sweetening agents in candies and sweets due to its intense sweetness (50 times sweeter than sucrose) and low number of calories. Due to the amphiphilic and chiral molecular structure, GA has anisotropic self-assembly behavior in water, which can first form long nanofibrils in water and then form supramolecular hydrogels with a 3D fibrillar network at a concentration over 0.3 wt% [15,19]. Our previous studies have reported that these self-assembled GA nanofibrils have superior interfacial activity and can be used as natural emulsifying and foaming agents for the fabrication of stable multiphase food systems (e.g., emulsions and foams) [15,20,21]. Furthermore, the spatially controllable assembly of GA nanofibrils at liquid interfaces and in the aqueous phase can lead to the simultaneous formation of fibrillar gel networks at the emulsion droplet surfaces as well as in the continuous phase, thus creating an O/W gel emulsion with excellent stability, stimulability, and processability. Compared to the commonly prepared gel-like high internal phase emulsions and cross-linked emulsion gels, the most prominent feature of our GA nanofibril-stabilized gel emulsions is the gelation mechanism, which mainly relies on the assembled fibrillar hydrogel network in the continuous phase, rather than the close packing of oil droplets or the crosslinking [15,22]. These findings show that food-grade GA and GA nanofibrils are very suitable building blocks for the construction of structured gel emulsions.

In view of the aforementioned great potential of dual-structured emulsions for diverse applications, there is no doubt that the development of dual-structured gel emulsions stabilized by GA nanofibrils can further enrich their properties and broaden their industrial applications. Accordingly, there is a need for an investigation of the effect of the addition of oil structuring agents on the formation and structural properties of the GA nanofibril-based gel emulsions. Monoglycerides (MG), one of the most promising oil-soluble emulsifiers and oleogelators, have been widely used in foods, cosmetics, and pharmaceuticals [23]. As an oleogelator, MG can self-assemble into an inverse lamellar phase with β-subcell packing, which further forms lamellar platelets and then generates a 3D crystalline network, entrapping liquid oil [24,25,26]. Herein, based on the assembly behaviors of GA and MG, we attempted to fabricate a novel dual-structured gel emulsion in which the continuous aqueous phase and the oil phase are structured by GA nanofibrils and MG crystals, respectively. We mainly investigated the impact of different MG concentrations on the formation and stability, structural properties, and thermoresponsive behaviors of the dual-structured gel emulsions. We characterized the microstructure and mechanical properties of gel emulsions by performing confocal microscopy, small deformation shear rheology, and large deformation mechanical tests. The applications of these dual-structured gel emulsions as food-grade encapsulation and delivery vehicles for various functional cargoes (i.e., *β*-carotene, vitamin B_12_, and limonene) were also evaluated. The results obtained are expected to expand the practical applications of dual-structured gel emulsions in food, cosmetics, and pharmaceutical products.

## 2. Results and Discussion

### 2.1. Formation of GA−MG Dual-Structured Gel Emulsions

**Figure 1:**
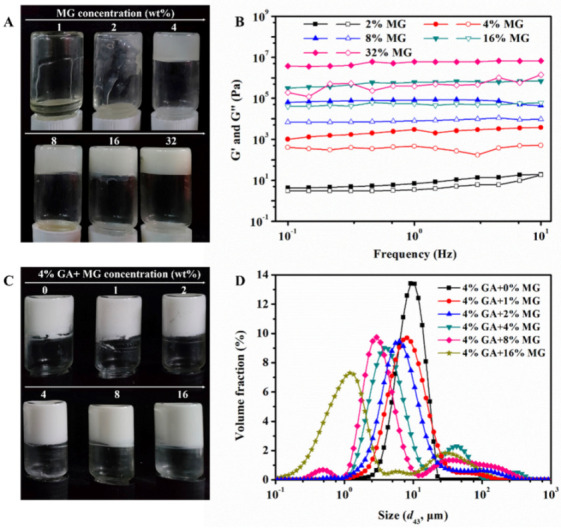
Photographs (**A**) and frequency sweeps (**B**) of MG oleogels with different MG concentrations (1–32 wt%). G′ and G″ are shown as filled and open symbols, respectively. Photographs (**C**) and droplet size distributions (**D**) of dual-structured gel emulsions containing 50 wt% sunflower oil prepared at a constant GA nanofibril concentration (4 wt%) and different concentrations of MG (0–16 wt%).

MG can self-assemble into a crystal structure in liquid oils and further form an oleogel with 3D crystalline networks at the temperature below its melting point [27]. Polarized light microscopy (PLM) was first used to observe the microstructure of MG oleogels prepared under different cooling conditions. As shown in Figure S1, all MG oleogels formed irregular needle-like crystals under PLM. Compared to the slow cooling at room temperature, the MG oleogels with rapid cooling formed some larger crystals. Figure S2A,B shows the elastic modulus (G′) and viscous modulus (G″) of these MG oleogels as a function of stress and frequency, respectively. At the same MG concentration, the oleogels with rapid cooling showed higher values of moduli than the samples fabricated by slow cooling, which suggests that the rapid cooling can enhance the mechanical strength of the MG oleogels, which might be related to the crystal formation rate (Figure S1). Considering the improved mechanical strength of oleogels, we therefore mainly prepared the MG oleogels by rapid cooling in the following sections. The impact of MG concentration on the formation and rheological properties of oleogels was then studied, and the results are shown in Figure 1A,B and Figure S3. In Figure 1A, the samples obtained at relatively low MG concentrations (0–2 wt%) can flow after inversion, whereas self-standing gels can be formed at higher MG concentrations (4 wt% and above), suggesting the formation of MG oleogels. Moreover, with increasing MG concentrations, the G′ and G″ values gradually increased, which indicates the promoted mechanical properties of the MG oleogels (Figure 1B and Figure S3).

Our previous studies have shown that the GA nanofibrils have controllable assembly at the oil−water interface and in the aqueous phase, which forms fibrillar networks on the surfaces of emulsion droplets as well as in the continuous phase, thus leading to the formation of gel emulsions [15,20,28]. Based on the self-assembly behaviors of GA and MG, we further prepared GA−MG dual-structured emulsions with a constant GA nanofibril concentration (4 wt%) and various MG concentrations (0–16 wt%). As can be seen, the obtained dual-structured emulsions displayed a homogeneous appearance (Figure 1C). Pure GA nanofibril-stabilized gel emulsion showed a mean droplet size (*d*_43_) at around 10 μm with a homogeneous size distribution (Figure 1D). Overall, the presence of MG in the oil phase led to a reduction in the *d*_43_ of emulsions, which may be related to the emulsifying ability of MG. In addition, compared to pure GA nanofibril-stabilized gel emulsions, the size distributions of those GA−MG dual-structured gel emulsions were significantly more polydisperse and broad, especially at higher MG concentrations (4–16 wt%, Figure 1D), indicating the occurrence of emulsion droplet aggregation probably due to the MG-induced oil phase structuring. XRD was further used to characterize the crystalline structures within the dual-structured emulsions. It can be observed that the diffractogram of all emulsion samples showed a hump-like peak at around 20° (Figure S4), which is mainly linked to the predominant amorphous structure of GA nanofibril-stabilized gel emulsions. Moreover, some sharp diffraction peaks were observed in the GA−MG gel emulsions with higher MG concentrations (4–16 wt%), which points to the presence of the crystalline structure of MG in the gel emulsions [13,26].

### 2.2. Microstructure of GA−MG Dual-Structured Gel Emulsions

**Figure 2:**
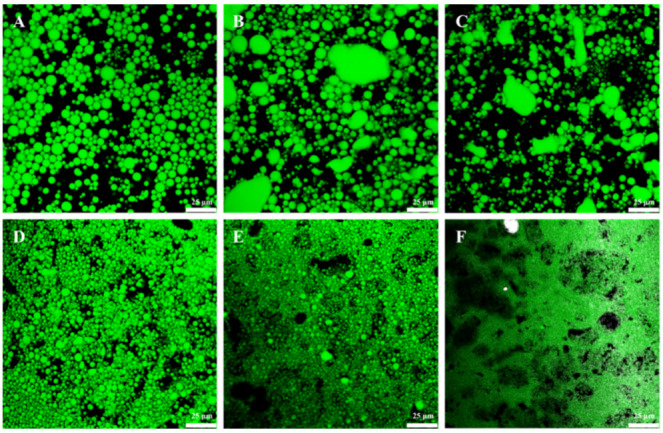
CLSM images of dual-structured gel emulsions containing 50 wt% sunflower oil prepared by 4 wt% GA nanofibrils and different MG concentrations: (**A**) 0, (**B**) 1, (**C**) 2, (**D**) 4, (**E**) 8, and (**F**) 16 wt%. The gel emulsions were prepared using oil dyed with Nile Red and oil droplets are green in the images. All scale bars are 25 µm.

**Figure 3:**
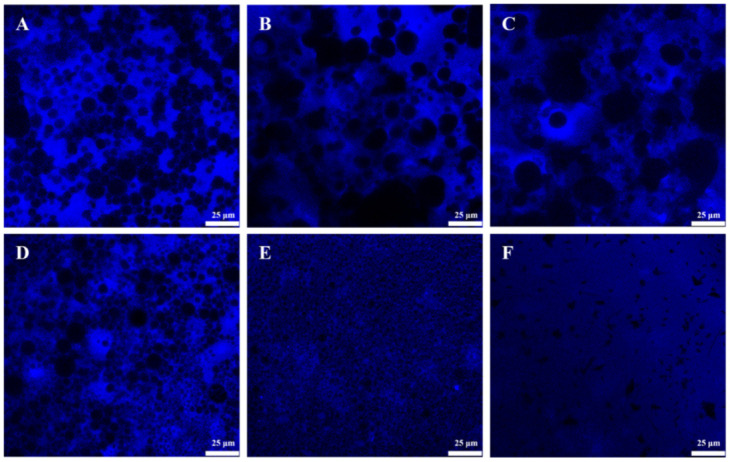
CLSM images of dual-structured gel emulsions containing 50 wt% sunflower oil prepared by 4 wt% GA nanofibrils and different MG concentrations: (**A**) 0, (**B**) 1, (**C**) 2, (**D**) 4, (**E**) 8, and (**F**) 16 wt%. The gel emulsions were prepared using the GA fibrillar network dyed with ThT, which is blue in the images. All scale bars are 25 µm.

To gain more insight into the structural properties of dual-structured emulsions, the microstructural observations of GA−MG gel emulsions were performed by using CLSM. Figure 2 and Figure 3 show the CLSM images of the GA−MG gel emulsions dyed with Nile Red and ThT, respectively. The dye Nile Red was used to label the oil phase of emulsions. As can be seen in Figure 2, compared to pure GA nanofibril-stabilized gel emulsions (without MG), the oil droplets of GA−MG gel emulsions, especially at higher MG concentrations (4–16 wt%), showed a smaller size and were packed more closely within the continuous matrix, in good agreement with the results of droplet size distribution (Figure 1D). Due to the specific binding to the fibrillar structure, the dye ThT was further used to label the GA fibrillar network of dual-structured gel emulsions [15,29]. As seen in Figure 3, the GA fibrillar network within the gel emulsions was clearly observed, and the size and distribution of oil droplets (black pores) were also consistent with the CLSM images in Figure 2. It is worth noting that at low MG concentrations (1–2 wt%), the gel emulsions showed obvious oil droplet aggregation, forming some large droplet agglomerates, which is probably due to the partial coalescence of oil droplets induced by the formation of MG crystals. In contrast, when the MG concentration was increased to 4 wt% or above, there was no obvious droplet aggregation in gel emulsions, which show a homogeneous microstructure with smaller and more closely packed droplets.

We speculated that the partial coalescence behavior of GA−MG dual-structured gel emulsions is related to the formation and structures of MG crystals in the oil phase which can affect the oil−water interfacial film. During the rapid cooling process, the larger and lamellar MG crystals were formed at relatively low MG concentrations, whereas small and uniform MG crystals were formed at higher MG concentrations (Figure S1). The different crystal structures and the interfacial activity of MG can thus efficiently tune the microstructure of dual-structured emulsions. At low MG concentrations (1–2 wt%), the oil−water interfacial film is mainly formed by GA nanofibrils. The larger and lamellar MG crystals near the oil−water interface may pierce the interfacial fibrillar film, thus inducing the formation of partially coalesced droplets. However, at higher MG concentrations (4 wt% or above), the interfacial film thickness can be enhanced due to the excellent interfacial activity of MG, which can lead to the formation of a bilayer shell of GA nanofibrils and MG crystals, thus efficiently inhibiting the interfacial film damage. Meanwhile, the smaller MG crystals formed in these smaller droplets may be insufficient to pierce the interfacial films. These features make those emulsion gels have a jamming structure with homogeneously distributed small emulsion droplets. Similar behaviors were observed in the emulsion gels with interior crystallizable droplets [5,30,31]. Based on the abovementioned results (Figure 1C,D, Figure 2 and Figure 3), we can conclude that the heterogeneous partial coalescence and jamming microstructures of the GA−MG dual-structured gel emulsions could be tuned by changing MG concentration.

### 2.3. Mechanical Properties of GA−MG Dual-Structured Gel Emulsions

**Figure 4:**
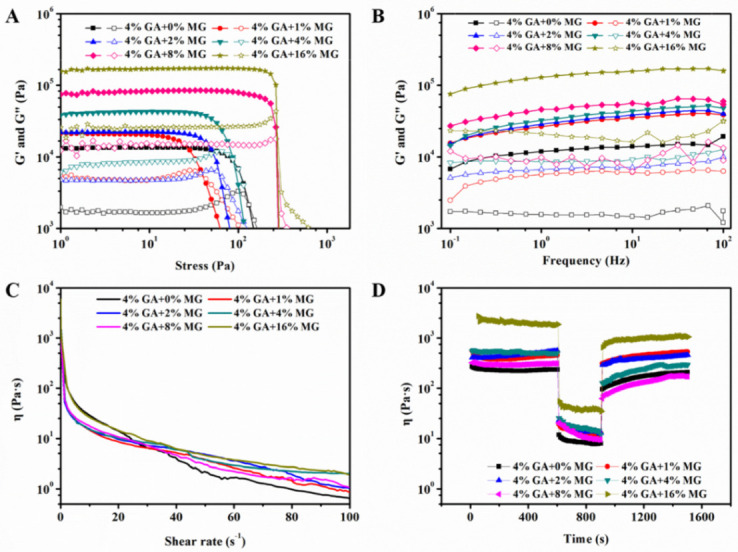
(**A**) Amplitude and (**B**) frequency sweeps for dual-structured gel emulsions prepared by 4 wt% GA nanofibrils and different MG concentrations (0–16 wt%). G′ and G″ are shown as filled and open symbols, respectively. (**C**) Viscosity curves and (**D**) thixotropic property, measured at alternating low and high shear rates (0.1 and 10 s^−1^, respectively), of these gel emulsions. All measurements were performed at 25 °C.

It is known that the droplet size and microstructure of emulsion gels can greatly affect their mechanical properties [2,32,33]. Herein, we determined the mechanical properties of the GA−MG dual-structured gel emulsions by performing a small deformation shear rheology and a large deformation compression test. Figure 4A,B shows the results of the oscillatory amplitude (stress = 1–1000 Pa, frequency = 1 Hz) and frequency (0.1–100 Hz, stress = 1 Pa, within the linear viscoelastic region, LVR) sweeps, respectively. As can be seen in Figure 4A, for all investigated samples, the elastic modulus (G′) was always higher than the viscous modulus (G″) within the LVR, indicating that the gel emulsions with different MG concentrations have elastic solid-like behavior. With increasing MG concentrations, the GA−MG gel emulsions showed significantly broader LVR, higher crossover stress (G′ = G″), and higher moduli values over the applied amplitude range, suggesting a higher gel strength. As shown in Figure 4B, the G′ and G″ values for all samples displayed a relatively weak frequency dependence. This indicates that the applied deformation rate does not significantly affect the rheological response of dual-structured gel emulsions. The loss tangent (G″/G′) values were always very low (below 0.1) over the entire frequency range, suggesting that these GA−MG gel emulsions have reasonably high gel strength. Figure S5 shows the hardness of the GA−MG gel emulsions with different MG concentrations obtained from the large deformation compression test. As can be seen, the hardness values increased with increasing MG concentrations, further indicating the enhanced mechanical strength of dual-structured gel emulsions. These results suggest that MG-induced oil phase structuring can significantly promote the mechanical performance of gel emulsions stabilized by GA nanofibrils.

Shear thinning and thixotropy behavior play a crucial role in practical applications of gel emulsions. Herein, the steady-state flow behavior of gel emulsions was measured by increasing shear rates from 0.1 to 100 s^−1^. As shown in Figure 4C, all dual-structured gel emulsions showed a prominent shear-thinning behavior. Compared to pure GA nanofibril-stabilized gel emulsions, these dual-structured emulsions showed a relatively higher viscosity value probably due to their stronger network structure (Figure 2 and Figure 3). Three interval thixotropic tests were further used to evaluate the structural recovery properties of the GA−MG dual-structured emulsions. In this test, viscosity was measured as a function of time under alternating cycles of low and high shear rates (0.1, 10, and 0.1 s^−1^). As can be seen in Figure 4D, all emulsion samples had a high viscosity value at an initial low shear rate of 0.1 s^−1^, and when the shear rate increased to 10 s^−1^, a drop in the viscosity values could be observed, illustrating structural damage. Upon decreasing the shear rate back to 0.1 s^−1^, the viscosity of dual-structured emulsions was increased again, suggesting the recovered structure. The structure recovery degree was calculated by comparing the maximum viscosity value in the third interval and the viscosity value at the end of the first interval [20]. The results showed that these GA−MG gel emulsions have good recovery behaviors with a relatively high recovery percentage of 57.1–88.8%.

### 2.4. Thermoresponsive GA−MG Dual-Structured Gel Emulsions

**Figure 5:**
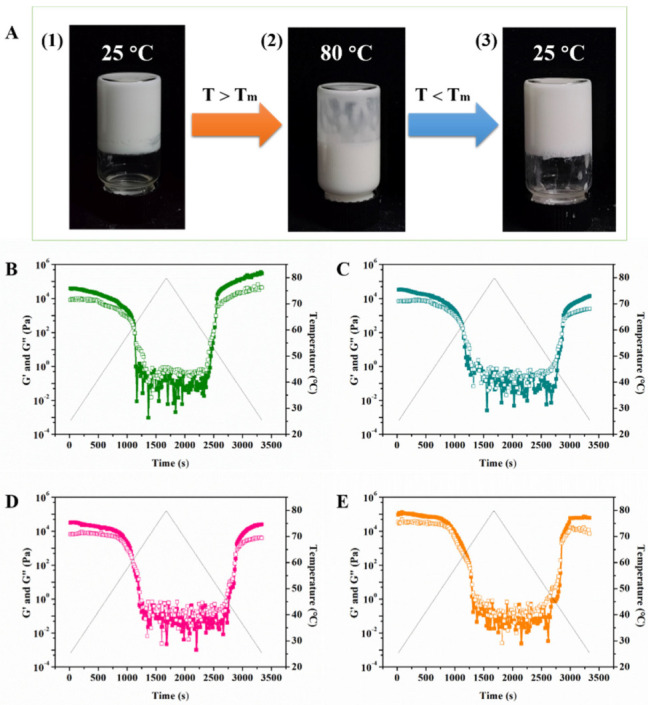
(**A**) Photographs showing the temperature-switchable behavior for a dual-structured gel emulsion with 4 wt% GA nanofibrils and 4 wt% MG: (**1**) Stable gel emulsion at 25 °C; (**2**) The sample was heated at 80 °C for 5 min, and then the gel emulsion became fluid; (**3**) Gelled emulsion was reformed by cooling the system to room temperature (25 °C). Storage modulus (G′) and loss modulus (G″) of dual-structured gel emulsions prepared by 4 wt% GA nanofibrils and different MG concentrations: (**B**) 2, (**C**) 4, (**D**) 8, and (**E**) 16 wt%, measured during the heating and cooling cycles. G′ and G″ are shown as filled and open symbols, respectively. All measurements were performed at 25 °C.

It is known that the GA supramolecular hydrogels have an interesting thermoresponsive behavior, showing a gel-sol transition temperature range of 55–60 °C, and above this temperature range, the hydrogen-bonding GA fibrillar network starts melting (T_m_) [15,20]. Our previous studies have shown that the reversible gel-sol phase transition behavior of the GA hydrogel network provides the possibility for the fabrication of GA nanofibril-based multiphase soft materials with interesting temperature-responsive behavior [15,16,20]. Herein, we further investigated the thermoresponsive properties of the GA−MG dual-structured gel emulsions (Figure 5). As can be seen from Figure 5A, the gel emulsion with 4 wt% MG was selected as an example to observe its thermoresponsive behavior. As expected, when the sample was placed in a water bath at 80 °C (above T_m_), the transition from a self-standing gel emulsion to the liquid emulsion was observed mainly due to the melting of the GA hydrogel network (Figure 5(A1,A2)). Upon decreasing the temperature to 25 °C (below T_m_), the gel emulsion could be obtained again, which is mainly attributed to the cooling-triggered regelatinization of GA nanofibrils in the continuous phase and around the droplet surfaces. Moreover, the reformation of the MG crystalline network into the oil phase during cooling also contributes to the recovery of the GA−MG gel emulsion. The same thermoresponsive phenomenon was observed in the gel emulsions with different MG concentrations (0–16 wt%). This confirms the thermoreversibility of the GA−MG dual-structured gel emulsions. This can be further supported by the results of temperature sweep tests (Figure 5B–E). As can be seen, the G′ and G″ values measured during temperature cycles for these GA−MG gel emulsions (2–16 wt% MG) showed a clear gel-to-sol transition during heating and a reversible gelation during cooling, suggesting the good thermoreversibility of dual-structured gel emulsions.

### 2.5. Cargo Encapsulation and Stability in Dual-Structured Gel Emulsions

**Figure 6:**
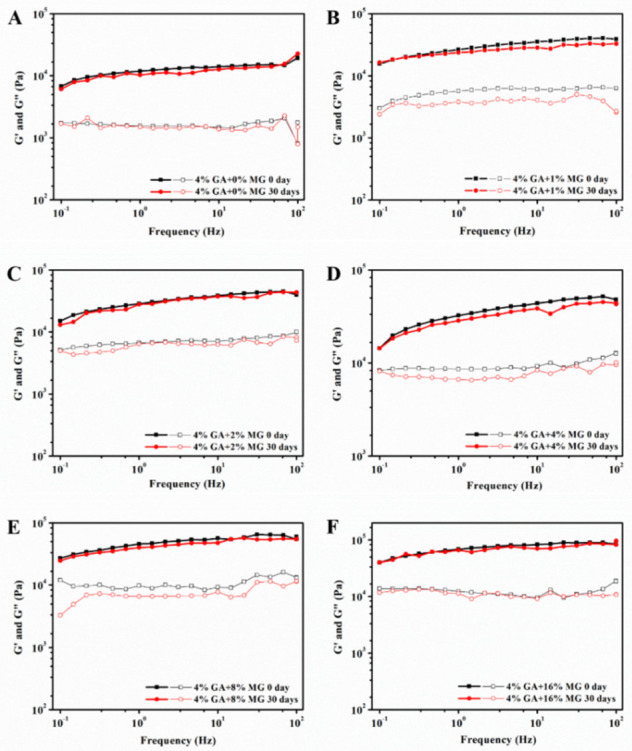
(**A**–**F**) Storage modulus (G′) and loss modulus (G″) as a function of frequency for dual-structured gel emulsions prepared by 4 wt% GA nanofibrils and different MG concentrations (0–16 wt%), measured at initiation (0 days) and after 30 days of storage at room temperature (25 °C). G′ and G″ are shown as filled and open symbols, respectively. All measurements were performed at 25 °C.

**Figure 7:**
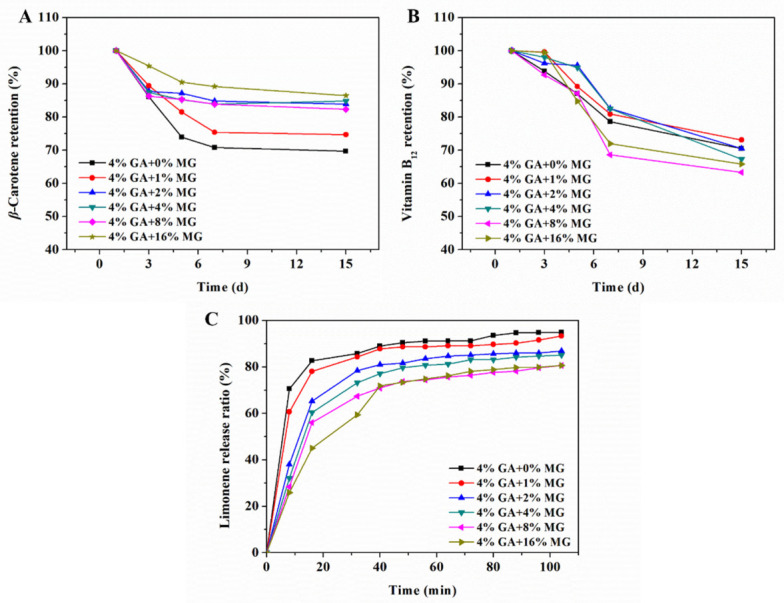
Retention of (**A**) *β*-carotene and (**B**) vitamin B_12_ in the GA−MG dual-structured gel emulsions during storage at room temperature 25 °C). (**C**) Real-time dynamic release profiles of limonene from gel emulsions at 50 °C.

In dual-structured emulsions, both the aqueous phase and the oil phase are structured, and the formed gel network structures can provide a strong barrier against adverse environmental factors and, thus, endow the emulsions with good stability [34]. We first adopted the frequency sweeps to evaluate the storage stability of the GA−MG dual-structured gel emulsions. As shown in Figure 6, the G′ and G″ values remained virtually unchanged after 30 days of storage, suggesting a good storage stability. This can be attributed to the fact that the multilayer GA fibril shell of emulsion droplets with high electrostatic forces can inhibit droplet coalescence [15,20]. Moreover, the structured oil phase by MG crystals also plays an important role in hindering oil droplet aggregation [35,36]. The ideal storage stability of these dual-structured gel emulsions based on GA nanofibrils, and MG crystals enables them to have more potential applications in protecting and delivering hydrophilic and hydrophobic actives. Therefore, we prepared the functional gel emulsions by dissolving *β*-carotene in the oil phase or vitamin B_12_ in the aqueous phase before the emulsification to evaluate the protective efficiency of these GA−MG gel emulsions. Figure 7A shows the retention of *β*-carotene encapsulated in the dual-structured gel emulsions during storage at room temperature (25 °C). Compared to pure GA nanofibril-based gel emulsion (without MG), the GA−MG gel emulsions can retain more *β*-carotene. It can be observed that only 70–75% of *β*-carotene was left in gel emulsions with 0–1 wt% MG, while the emulsion gels with 2–16 wt% MG could retain about 80–85% of *β*-carotene. The high retention of *β*-carotene for the emulsions with higher relative MG concentrations is mainly attributed to the more uniform and denser network of gel emulsions, which can retard the diffusion of pro-oxidants or free radicals and, thus, protect *β*-carotene from degradation and oxidation.

Figure 7B shows the retention of vitamin B_12_ in the gel emulsions during storage at 25 °C. As can be seen, in all cases, over 60% of the original content of vitamin B_12_ could be retained after 15 days of storage. Compared to the gel emulsions with relatively low MG concentrations (0–1 wt%), the samples with 2–4 wt% MG exhibited a better protective effect after 3 days of storage, which may be related to their partial coalescence structure. It should be noted that the dual-structured emulsions with higher MG concentrations (8–16 wt%) exhibited a decreased protective efficacy. This could be attributed to the fact that these gel emulsions with 8 and 16 wt% MG had a smaller relative droplet size and, thus, a higher surface area, which may have accelerated the oxidation and degradation of vitamin B_12_ [37]. The results above indicate that GA−MG dual-structured gel emulsions with good storage stability can be developed as effective delivery vehicles for the encapsulation and protection of hydrophobic and hydrophilic cargoes (e.g., *β*-carotene and vitamin B_12_).

### 2.6. Controlled Limonene Release in Dual-Structured Gel Emulsions

The dual-structured emulsions represent a promising strategy for the provision of controlled flavor release because they have the capacity to efficiently load hydrophilic and hydrophobic volatile compounds as well as display sustained aroma perception. Herein, the GA−MG dual-structured gel emulsions were further tested for their ability to control the release of a volatile lipophilic aroma compound (D-limonene). Figure 7C presents the dynamic release profile of limonene from the dual-structured gel emulsions. As can be seen, for all samples, limonene showed a continuous and rapid speed release during the initial 32 min, followed by a slower release rate. In comparison with pure GA nanofibril-stabilized gel emulsion, the dual-structured emulsions exhibited an obviously slower limonene release content and release rate. Further, with increasing MG concentration, the release of limonene from these gel emulsions gradually became slower. This can be explained by two factors: (1) The increase in MG concentration can enhance the crystalline network in the oil phase, which thus provides more barriers to limonene diffusion; (2) the good interfacial activity of MG crystals enables them to adsorb at the oil−water interface, strengthening the interfacial film and, thus, preventing limonene diffusion from the oil phase to the aqueous phase. Based on the above results and analyses, it can be concluded that the GA−MG dual-structured gel emulsions are novel and efficient aroma delivery systems that can improve the flavor perception of foods, and their volatile release can be well controlled by simply changing the MG concentration, i.e., the extent of oil phase structuring.

## 3. Materials and Methods

### 3.1. Materials

Glycyrrhizic acid mono ammonium salt (GA; purity > 98%) was purchased from Acros Organics (New Jersey, NY, USA). Monoglycerides (MG; 99% purity), vitamin B_12_ (98% purity), natural *β*-carotene (purity ≥ 96%), and (R)-(+)-limonene (purity > 99%) were provided by Aladdin (Shanghai, China). Nile Red and thioflavin T (ThT) were purchased from Sigma-Aldrich (St. Louis, MO, USA). Sunflower oil was purchased from a local supermarket (Guangzhou, China).

### 3.2. Preparation of Monoglyceride (MG) Oleogels

MG powders were weighed and then completely dissolved in sunflower oil at 80 °C under magnetic stirring. The resulting solutions were transferred to icy water (4 °C, rapid cooling) or room temperature (25 °C, slow cooling) to promote gelation and the formation of oleogels. Oleogels with different MG fractions, i.e., 2%, 4%, 8%, 16%, and 32% (*w*/*w*), were prepared.

### 3.3. Preparation of GA−MG Dual-Structured Gel Emulsions

GA powder was dispersed in Milli-Q water by heating at 80 °C under magnetic stirring to prepare a stock dispersion of GA nanofibrils. The dual-structured emulsions were prepared by first adding the hot MG solution (80 °C) with different concentrations (0, 2, 4, 8, 16, and 32 wt% in oil, *w*/*w*) into GA nanofibril solutions under mild agitation for 2 min. Then, the resulting dispersions were immediately sheared using an Ultra-Turrax T10 (IKA-Werke GmbH & Co., Staufen, Germany) at 20,000 rpm for 2 min. The resultant emulsions were rapidly cooled in an ice bath (4 °C) or slowly cooled at room temperature (25 °C) to promote the gelation of both phases and the subsequent formation of dual-structured gel emulsions. The prepared samples were stored at room temperature (25 °C) before further experiments. The final GA nanofibrils concentration was 4 wt%, and the final MG concentration was 0, 1, 2, 4, 8, and 16 wt%. The preparation of functional dual-structured gel emulsions was performed similarly by dissolving *β*-carotene/limonene (0.1 wt% of oil) into the oil phase or vitamin B_12_ (0.1 wt% of water) into the water phase before the homogenization step.

### 3.4. Droplet Size Measurements

The droplet size of the dual-structured emulsions was measured using a Mastersizer 3000 (Malvern Instruments Ltd., Malvern, UK) after appropriate dilution with water. The refractive indices of sunflower oil and water were taken as 1.474 and 1.330, respectively. All measurements were carried out at 25 °C, and the results reported are averages of the three measurements.

### 3.5. Polarized Light Microscopy (PLM)

The microstructure of MG oleogels was studied using a polarized light microscope (PLM, Axioskop 40 Pol/40A Pol, ZEISS, Göttingen, Germany) equipped with a Power Shot G5 camera (Canon, Tokyo, Japan) and a Liakam hot stage (CI 94). For PLM observation, the samples were placed on a flat slide and covered by a coverslip. The magnification was 500× (50 × 10) and each image was acquired under polarized light. Polarized light was used to observe the distribution and structure of the MG crystals in oleogels.

### 3.6. Confocal Laser Scanning Microscopy (CLSM)

The microstructure of dual-structured emulsions with different MG concentrations was visualized using a confocal laser scanning microscope (CLSM, Leica Microsystems Inc., Heidelberg, Germany). For CLSM visualization, Nile Red (0.1 wt%, a fluorescent dye for oil) was first dissolved in sunflower oil, which was used to fabricate the dual-structured emulsions. The obtained emulsions were placed in the center of the concave slide and then gently covered with coverslips. They were observed using an argon krypton laser (ArKr, 488 nm) at room temperature (25 °C). ThT was used to label the GA fibrillar network of dual-structured emulsions. ThT (0.01 wt%) was first dissolved in GA solutions before the preparation of emulsions. An argon krypton laser (458 nm) with a 470–560 nm emission fluorescence was used for ThT-stained samples. The oil phase dyed with Nile Red was green, and the GA fibrillar network dyed with ThT was blue.

### 3.7. Rheological Measurements

The rheological measurements of MG oleogels and GA−MG dual-structured gel emulsions were investigated using a HAAKE MARS60 rheometer (HAAKE Co., Vreden, Germany) equipped with a Universal Peltier system and a water bath (HAAKE A40) for temperature control. A parallel plate geometry of 35 mm diameter with a gap of 1.0 mm was used. Approximately 2 g of the sample was placed on the surface of the rheometer plate for the test. A range of oscillatory experiments including an amplitude sweep (stress = 1–1000 Pa, frequency = 1 Hz) and a frequency sweep (frequency = 0.1–100 Hz, stress = 1 Pa, within the linear viscoelastic region) were performed at 25 °C. The temperature sweep test was carried out by heating from 25 to 80 °C and cooling back to 25 °C at a rate of 2 °C/min at a constant stress of 10 Pa and a frequency of 1 Hz. For all the aforementioned measurements, the elastic modulus (G′) and viscous modulus (G″) of oleogels and gel emulsions were recorded. For flow measurements, the samples were subjected to increasing shear rates from 0.01 to 100 s^−1^ at 25 °C. For thixotropy evaluation, the viscosity of dual-structured gel emulsions with time was measured at alternating shear rates (0.1 and 10 s^−1^, respectively).

### 3.8. Large Deformation Mechanical Properties

The large deformation compression test of dual-structured gel emulsions was performed using an Instron 5943 universal testing machine (Instron, Norwood, MA, USA) equipped with a 0.1 N load cell and a cylindrical probe of 25 mm. For the test, all samples with a diameter of 22.3 mm were compressed at a rate of 10 mm/min to 60% strain and then the relative displacement was recorded. All the measurements were performed at 25 °C.

### 3.9. XRD Analysis

Crystalline structures in dual-structured gel emulsions were evaluated using a multi-position automatic injection X-ray diffractometer (X′ pert Powder, PANalytical, Malvern, UK). The XRD experiments were performed with a Cu source, X-ray tube at 40 kV and 40 mA. The angular scanning was done in the 2θ range of 5–60° at a scanning rate of 12° per min, and data were collected with the step of 0.013°.

### 3.10. Cargo Stability of GA−MG Dual-Structured Gel Emulsions

The storage stability of dual-structured emulsions was first illustrated by comparing the frequency curves of samples stored at 25 °C for 0 and 30 days. To evaluate the stability of cargoes (*β*-carotene and vitamin B_12_) encapsulated into gel emulsions, the cargo-loaded dual-structured emulsions were stored at 25 °C for 1, 3, 5, 7, and 15 days, and then the cargo content was determined. For *β*-carotene, the determination of its content in gel emulsions over time followed the method of Tan with some modifications [38]. In detail, the gel emulsions (0.2 g) were extracted with 9.8 g of a mixture of ethanol and hexane (2:3, *v*/*v*). After shaking, the hexane phase was collected and appropriately diluted with hexane, and then determined at 450 nm using a UV-visible spectrometer (C40 Touch, IMPLEN, Munich, Germany). The concentration of *β*-carotene was calculated by referring to a standard concentration-absorbance curve under the same condition. The retention percentage of *β*-carotene was expressed as retention (%) = C/C_0_ × 100%, where C_0_ was the initial content of *β*-carotene and C was the content of *β*-carotene at different storage times.

The content of vitamin B_12_ in gel emulsions over time was measured using the method by Li with certain modifications [39]. In detail, at certain time intervals, 0.4 g of gel emulsions was added into 9.6 g of ethanol, followed by a mild stirring. The resulting dispersion was then centrifuged at 10,000 rpm for 15 min to acquire the supernatant. The concentration of vitamin B_12_ in the supernatant was measured at 361 nm. The retention percentage of vitamin B_12_ was calculated as retention (%) = W/W_0_ × 100%, where W_0_ was the initial content of vitamin B_12_ and W was the cargo content after storage.

### 3.11. Limonene Release of GA−MG Dual-Structured Gel Emulsions

The dual-structured gel emulsions were individually transferred to 20 mL headspace vials (silicone/PTEF seals) immediately after homogenization, and the vials were kept sealed at 4 °C to avoid volatile losses. For aroma release analysis, a simulated nose breath device was used, which was composed of a cylindrical glass cuvette (55 mm in diameter and 285 mm in height) sealed with a cap and a constant temperature circulation water bath with the temperature controlled at 50 °C [40,41,42]. Injections of the headspace (1 mL) were conducted using a 2.5 mL thermostated gas-tight syringe (Hamilton, Bonaduz, Switzerland) on an Agilent 7890B gas chromatograph (GC) equipped with an HP-5 column (30 m, 0.32 mm i.d., film thickness 0.25 μm) and coupled with a flame ionization detector (FID) (Agilent Technologies, Santa Clara, CA, USA). Headspace vials containing gel emulsions were incubated at 50 °C every 8 min and injected into the GC using a preheated 2.5 mL thermostatic gastight syringe (Hamilton, Bonaduz, Switzerland) PAL LHX-xt (Zwingen, Switzerland) for online analysis. The temperature of the GC Injector and FID was 250 °C. The rate of injection was 200 μL/s. The helium carrier gas velocity was 1 mL/min. To quantify the concentrations of limonene in the headspace, a gas calibration curve was plotted using peak areas obtained from GC analysis against the known concentrations of limonene. The release of limonene was analyzed according to the above GC method. Limonene release percentage in dual-structured gel emulsions was calculated as the following equation: Limonene release ratio (%) = W/W_0_ × 100%, where W_0_ and W were the initial content of limonene and the content of limonene at different times, respectively. The results were expressed as the mean value of the triplicate analyses.

### 3.12. Statistical Analysis

The data from all the experiments were expressed as the average ± standard deviation. Statistical comparison was performed by analysis of variance (ANOVA) using the SPSS 19.0 statistical analysis software (IBM, Armonk, NY, USA). Duncan’s test was used to compare mean values among samples at different strain and frequency treatments utilizing a level of significance of 5%.

## 4. Conclusions

In this work, we successfully developed a novel dual-structured gel emulsion by incorporating monoglyceride (MG)-based oil phase structuring into the emulsions stabilized by glycyrrhizic acid (GA) nanofibrils. The formation of GA fibrillar networks in the aqueous phase and MG crystalline networks in the oil phase led to the formation of the dual-structured gel emulsions. We investigated the effects of MG concentration on the formation and stability, structural properties, and thermoresponsive and cargo release behaviors of the GA−MG gel emulsions. The microstructure of the dual-structured gel emulsions is found to be largely dependent on the concentration of MG in the oil phase. At low MG concentrations (1–2 wt%), the larger and lamellar MG crystals were formed, which may pierce the interfacial fibrillar film and, thus, induce the formation of partially coalesced droplets. In contrast, at high MG concentrations (4 wt% or above), the smaller MG crystals with enhanced interfacial activity can lead to the formation of a bilayer shell of GA nanofibrils and MG crystals, which can inhibit interfacial film damage and, thus, form a jamming structure with homogeneously distributed small emulsion droplets. The GA−MG dual-structured gel emulsions showed significantly improved mechanical performance as well as good thermoreversibility. These food-grade GA−MG gel emulsions can be used as stable delivery vehicles for the encapsulation and protection of hydrophobic and hydrophilic bioactive cargoes (*β*-carotene and vitamin B_12_). In addition, they also displayed the potential as efficient aroma delivery systems (e.g., limonene), showing a highly controlled volatile release by simply changing MG concentration. We expect that this dual-structured emulsion strategy can broaden the promising applications of natural saponin GA-based gel emulsions in the food, pharmaceutical, and personal care product industries.

## Data Availability

Not applicable.

## Supplementary Materials

The following supporting information can be downloaded at: https://www.mdpi.com/article/10.3390/molecules27196542/s1, Figure S1: PLM images of MG oleogels with different MG concentrations; Figure S2: Amplitude and frequency sweep for MG oleogels; Figure S3: Stress amplitude for MG oleogels with different MG concentrations; Figure S4: XRD diffractograms of dual-structured gel emulsions; Figure S5: Large-deformation compression of dual-structured gel emulsions.
