# Supplementary material for: Thermoresponsive Dual-Structured Gel Emulsions Stabilized by Glycyrrhizic Acid Nanofibrils in Combination with Monoglyceride Crystals
Source: Molecules. 2022 Oct 3;27(19):6542. doi: 10.3390/molecules27196542 (PMC9573016; doi:10.3390/molecules27196542)
Supplement: Supplementary file 1 [file molecules-27-06542-s001.zip › molecules-1942366-supplementary.pdf]

Supporting Information

Thermoresponsive Dual-Structured Gel Emulsions Stabilized by Glycyrrhizic Acid Nanofibrils in Combination with Monoglyceride Crystals

Jialing Chen [1], Qing Li [1], Ruijie Du [1], Xinke Yu [1], Zhili Wan [1,2,*] and Xiaoquan Yang [1]

[1] Laboratory of Food Proteins and Colloids, School of Food Science and Engineering, Guangdong Province Key Laboratory for Green Processing of Natural Products and Product Safety, South China University of Technology, Guangzhou 510640, China

[2] Overseas Expertise Introduction Center for Discipline Innovation of Food Nutrition and Human Health
(111 Center), Guangzhou 510640, China

* Correspondence: zhiliwan@scut.edu.cn; Fax: +86-20-8711-4263

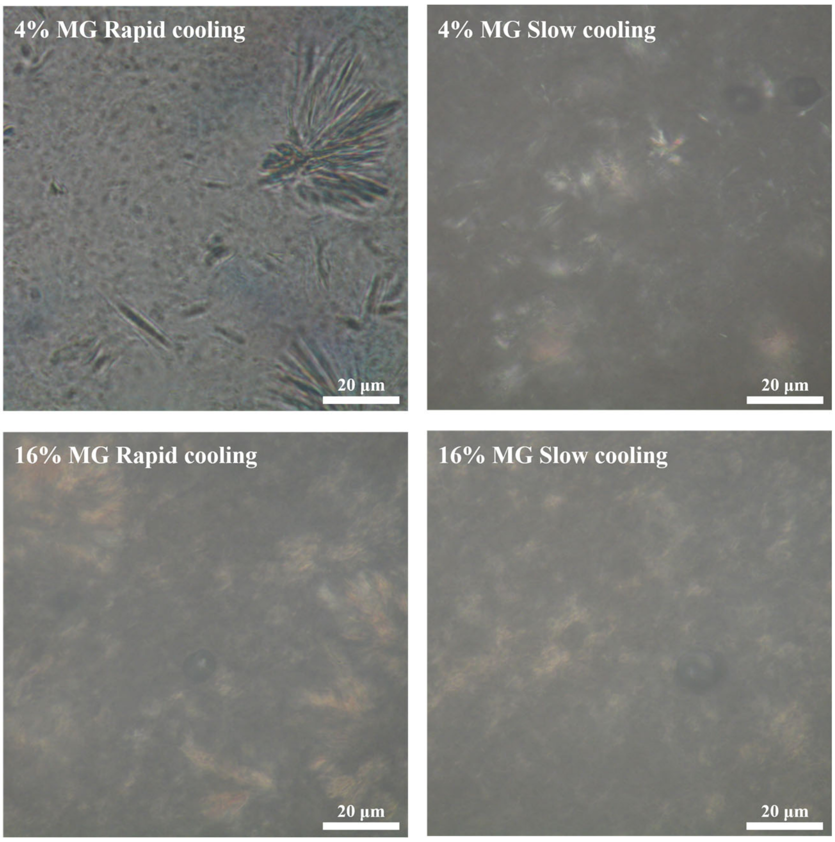

Figure S1. PLM images of MG oleogels with different MG concentrations (4 and 16 wt%), obtained at different cooling conditions (rapid cooling at 4 °C and slow cooling at 25 °C). Scale bar: 20 µm.

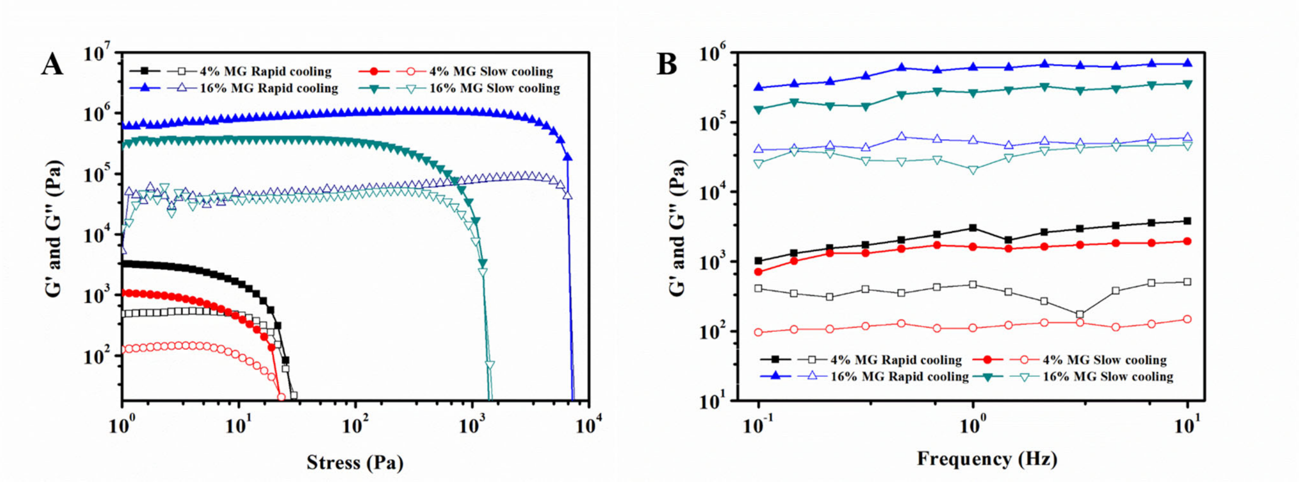

Figure S2. (**A**) Amplitude and (**B**) frequency sweep for MG oleogels (4 and 16 wt%), obtained at different cooling conditions (rapid cooling at 4 °C and slow cooling at 25 °C). G′ and G″ are shown as filled and open symbols, respectively. All measurements were performed at 25 °C.

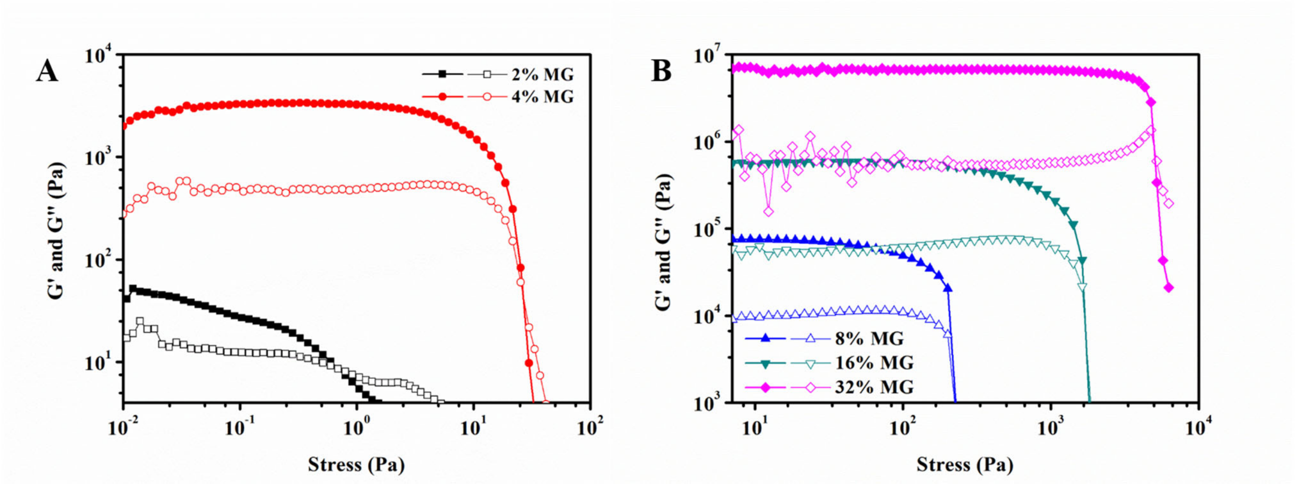

Figure S3. Stress amplitude for MG oleogels with different MG concentrations (2–32 wt%). G′ and G″ are shown as filled and open symbols, respectively. All measurements were performed at 25 °C.

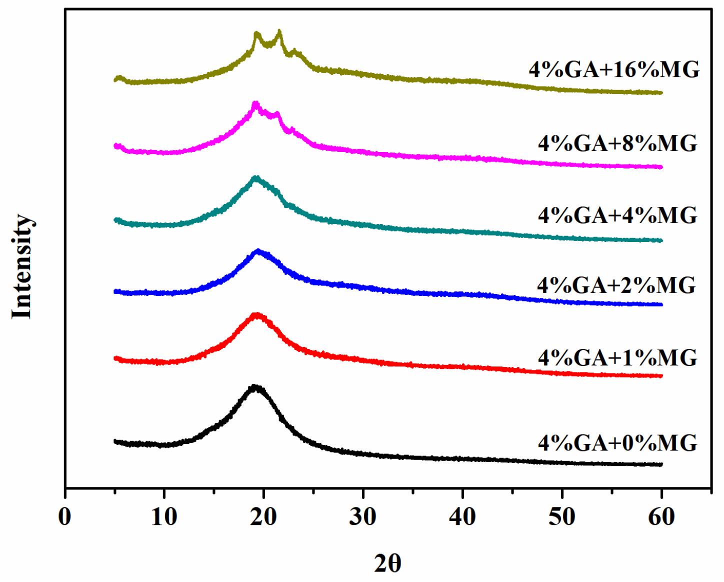

Figure S4. XRD diffractograms of dual-structured gel emulsions prepared by 4 wt% GA nanofibrils and different MG concentrations (0–16 wt%).

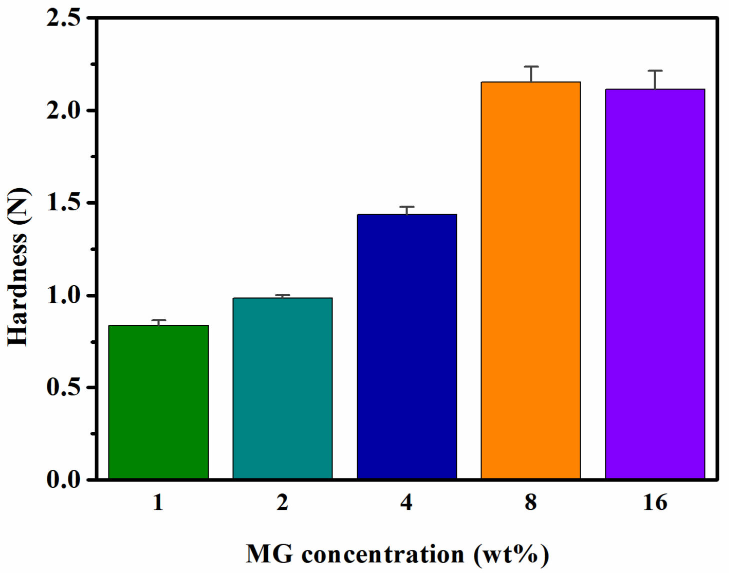

Figure S5. Large-deformation compression of dual-structured gel emulsions prepared by 4 wt% GA nanofibrils and different MG concentrations (0–16 wt%). All measurements were performed at 25 °C.
